# Peer champions responses to nudge-based strategies designed to reduce prolonged sitting behaviour: Lessons learnt and implications from lived experiences of non-compliant participants

**DOI:** 10.3934/publichealth.2022040

**Published:** 2022-07-12

**Authors:** P. Dean Cooley, Casey P. Mainsbridge, Vaughan Cruickshank, Hongwei Guan, Anjia Ye, Scott J. Pedersen

**Affiliations:** 1 College of Arts, Law and Education, University of Tasmania, Launceston, Tas, Australia; 2 Ithaca College, Ithaca, New York, USA

**Keywords:** nudge theory, prolonged sitting, sedentary behaviour, office workers, gender work roles

## Abstract

Occupational sedentariness is problematic for office-based workers because of their prolonged sitting periods and the advent of technology which reduces work-based movement. A common workplace strategy to deal with this preventable health risk is to have workers engage in brief movement breaks throughout the workday. To date, the use of interventions underpinned by individual self-regulation has had less than optimal impact on changing workers sedentary work behaviours. An alternative design for workplace interventions is the use of nudge theory. Nudge theory incorporates strategies that are delivered at the point of choice designed to influence individual decision making regarding alternative behaviour options. In this study, desk-based workers were exposed to two nudge strategies which suggested alternative behaviours of regular standing and taking movement breaks during work periods to the default behaviours of prolonged sitting and sedentary work behaviour. A small group of women managers who served as peer champions (n = 6), withdrew early from the study, and then took part in an exit interview to gain an understanding of their experiences of being exposed to the two nudge strategies. Verbatim transcripts were analysed using inductive, reflexive thematic analysis. Two major themes with seven second order themes central to their experiences were extracted: facilitative behaviour and feelings (advocacy, acceptance & facilitative burden) and dysfunctional behaviours and feelings (dysfunctional behaviour & feelings, control, reactance & presenteeism). Participants initially perceived a positive exchange associated with exposure to nudge strategies. Yet, participants' emotional connection to their work roles and behaviour were perceived as a negative exchange. Participants cited numerous maladaptive feelings because of a perception of incongruency with the established work normative behaviour. These findings reveal that nudge strategies of reduced choice and social norms are viable, but perceptions of monitoring can moderate adherence.

## 1. Introduction

Desk-based workers consistently report that they sit for up to 10 hours per day to perform tasks at the office or when working from home [1],[2]. Occupational prolonged sitting behaviour is a public health concern [3] because it contributes to overall levels of sedentariness [4], establishes a pattern of sedentary behaviour that is replicated in leisure time activity [5],[6], and is associated with a range of adverse health outcomes [7]. Behaviour remains a critical determinant of health [8] and as the physical and social work environments contribute to habitual work behaviour [9], a key strategy in workplace health interventions (WHI) is changing individual work behaviours to incorporate increased amounts of movement [6],[10]. To date, most WHI have focused on self-regulated designs which allow workers individual agency in determining their behaviour choices such as deciding to use a standing desk instead of sitting [11], taking walking breaks [12], or standing during meetings [13],[14]. Evident in the literature is the fallibility of behaviour-change designs which reflect individual agency over structural influences [9],[15],[16]. This fallibility is connected in part, to the heuristics and biases associated with decision making [17]. To date, the application of a sit less, move more strategy in WHI has not embraced alternative theoretical behaviour change models. One model that offers potential moderation of the heuristic and bias effects known to occur in self-regulatory decision making environments is nudge theory [17].

Nudge theory outlines a number of strategies that potentially offer individuals cues to consider alternative choices to their normal behaviour choice [17]. These strategies include reducing choice options, anchoring, prompts, and social norms [18]. The intent of a nudge is not to change behaviour to the extent of changing a decision to the opposite of what would have been selected, but prompt individuals to evaluate alternative behaviour options before making a choice [8]. For example, the use of message prompts which suggest healthier food options at the point of choice, can trigger individuals to choose the alternative healthier food options instead of their normal default food choices [19]. To date nudge theory has been successfully applied in a wide variety of situations [18], but its use in workplaces to change entire workforce behaviour choices to decrease occupational sedentariness is limited to a handful of studies [20]–[22].

These studies were implemented across several large work organisations and used a combination of passive choice and social norms nudges [23],[24] to successfully have desk-based workers choose the alternative behaviours of regularly standing and participating in non-exercise physical activity instead of the default choices of remaining seated and sedentary during work. Nonetheless, the evaluation was restricted to physiological and compliance variables [25],[26]. Some data [25] suggested variability in the success of the nudges to alter individual choice, but there was no investigation of potential individual or situational moderating variables which might have interacted with the selected nudges. These data are critical because behaviour change is open to many exchanges which may or may not moderate decision-making and subsequently reduce the effect of the nudge strategy [27]. For example, one study using a message prompt to try and nudge workers to choose standing rather than sitting during work meetings [13] reported one exchange outcome was an increase in some workers' perceptions of self- and others- monitoring of their alternative behaviour choice [28],[29]. Consequently, some workers experienced negative affect (i.e., anxiety) because they feared that choosing the alternative behaviour was perceived with being incongruent with workplace and work role expectations. Consequently, for some workers, this sequala moderated their effect of the nudge so that they altered their choices to reflect the default behaviour.

The current study therefore aims to gain a more nuanced understanding of the lived experiences of participants who withdrew from a nudge-based WHI. In particular, the aim of the study was to explore what exchanges managerial participants perceived to have occurred when they acted as peer champions (PC) for a WHI which used a combination of passive choice architecture and norm-based strategies. Our research question was “what are the lived experiences of managerial desk-based workers who have withdrawn from a nudge-based WHI designed to decrease prolonged sitting time?” This paper represents one part of a larger project that aims to develop nudge-based interventions to reduce occupational sedentariness.

## 2. Materials and methods

### 2.1. Design

As there were no previous published data for nudge-based WHI designed to reduce prolonged sitting, an inductive, reflexive thematic analysis [30] provided flexibility to allow the data to determine the emerging themes. A criticism of this approach is that such flexibility in the construction of themes can result in researchers missing nuanced experiences [31]. Nonetheless, the researchers agreed that the small sample size would mitigate this risk [32].

### 2.2. Participants

The sample was a purposive sample comprised of women office managers who had withdrawn from the larger study sited in a large governmental multisite organisation (N = 250). The participants were part of a group of office managers (N = 27; 18 = women, 9 = men) who had responded to email and internal newsletters to host the study in their respective work groups within the larger study and volunteered to act as PC for the study. Participants had supervisory responsibility for various sized groups of workers (Table 1). Within five weeks of the initiation of the intervention, some participants withdrew from the larger study (n = 6). No other managerial PC withdrew from the study. Given that the withdrawn participants had requested that their respective work teams become part of the study, had volunteered to be PC, comprised 22 per cent of the managerial PC sample, and were all women, the researchers decided that they would potentially provide unique lived experience perspectives of the exchanges perceived from being involved in a nudge-based WHI. Exploring these possible exchanges would provide insight into possible individual and structural moderating variables. The researchers emailed all withdrawn PC with a request to participate in an exit interview. All agreed and thus formed the purposive sample for this study.

**Table 1.** Participants' characteristics, workplace descriptors & interview times.

Pseudonym	Site & Days in WHI	Age	Gender	Highest Qualification	Job Status Role & Years Exp^a^	Supervisory Role^b^	1st Meeting Type & Interview Time (min: sec)	2nd Meeting Type & Interview Time (min: sec)	Total Time
Fiona	A 14 days	44	Woman	Degree	F/T Team leader 4 yrs	Small	Individual F2F 45:23	Individual Telephone 24:30	69.53
Colleen	A 12 days	38	Woman	Technical certificate	F/T Team leader 6 yrs	Medium	Individual F2F 45:21	Individual Telephone 31:20	76.41
Heidi	B 18 days	42	Woman	Professional	F/T Team leader 7 yrs	Small	Individual F2F 89:10	Individual Telephone 25:15	114.2
Karen	C 21 days	37	Woman	Technical	F/T Team leader 5 yrs	Medium	Individual F2F 43:23	Individual Telephone 15:21	58.44
Jane	E 19 days	38	Woman	Degree	F/T Team leader 4 yrs	Medium	Individual F2F 39:50	Individual Telephone 24:15	63.55
Doris	D 22 days	42	Woman	Degree	F/T Team leader 8 yrs	Medium	Individual F2F 58:03	Individual Telephone 23:50	81.53

Note: ^a^F/T = Fulltime, F2F = Face-to-face; ^b^Small = 5–10 staff, Medium = 10–15 staff, Large = 15–20 staff.

### 2.3. The intervention

The intervention design for the larger study replicated a previous design [21]. The intervention was delivered through a web-based desktop application. The software, which could not be deactivated by participants, initiated at first, a timed electronic nudge which provided participants with information that they had been sitting for 45 minutes and they should stand up to engage in a movement break [33],[34]. The timing of the initiation was based on biological data that indicates that after 45 minutes of sitting, the body stops production of protective cardiovascular enzymes [7]. At this point of choice, participants could voluntarily decide to participate by pressing the space bar on their keyboards. This action activated a stand up and move screen which contained a selection screen of work-appropriate, non-exercise physical activity movement breaks such as walking the stairs, or standing while making a phone call [35]. Upon completion of the movement break, participants entered their movement activities via the screen portal, had their previous work screens returned, and recommenced their work routines. If participants chose not to respond to the first nudge, a second nudge was delivered 15 minutes later. This nudge reflected passive choice architecture whereby participant's computer screens were automatically replaced with the movement break selection screen. Participants then followed the same routine as those who voluntarily decided to stand and move. To support the behaviour change process, the design incorporated work managers across sections of the organisation to act as voluntary PC to model the new health behaviour during work [36]. PC completed a two-hour training session with a curriculum content of; education about the health concerns associated with prolonged sitting and sedentary behaviour, the design of the intervention, use of the software and time for PC to ask clarifying questions. All training took place in two venues on consecutive days. PC acting as role models for new workplace behaviour can facilitate and enhance social support and acceptance for new workplace behaviours [36]. Moreover, PC enhance fellow workers' feelings of efficacy associated with maintaining new behaviour [10], thus mitigating feelings of negative affect [36].

### 2.4. Procedures and interview schedule for this study

One full-time male Australian academic (VC) with a PhD and extensive interviewing experience in kinesiology-based research, who was independent of the development and implementation of the WHI and unknown to participants conducted all interviews to ameliorate any potential sensitivity issues associated with participant withdrawal [37]. Due to the multisite work structure, interviews took place during participants' scheduled work breaks, thus necessitating two interviews to enable participant's an opportunity to provide data to all questions. Interviews were scheduled to occur 3–6 (*Mweeks* = 4.3 ± 1.5) weeks after participants' withdrawal notifications with the second interviews occurring 2–3 months (*Mweeks* = 11.7 ± 2.1) afterwards. This schedule reflected a balance decision between participants and researchers, to ensure minimal disruption to the organisation's operations [38]. No repeat interviews occurred. All first interviews took place at each participant's place of employment in a private area not accessible to other staff, with only the interviewer present. A subsequent video interview took place at agreed times and locations. All interviews were digitally recorded with field notes taken by the interviewer that noted his experiences of conducting the interviews, his impressions, and possible assumptions about the participants, as well as any relevant visual details. The interviewer explained that the study was about participants experiences of the intervention before beginning with the interview questions. All recorded data were transcribed using NVIVO software (N = 467.23 minutes), collated in MSExcel, and transferred to MSWord. All transcripts were de-identified; all names were replaced with pseudonyms and identifying characteristics were removed or changed. To ensure comparability, interviews were carried out using a discussion guide to allow for triangulation of data to improve validity [30]. Questions were semi-structured and based on previous self-regulatory WHI lived experience data (Supplementary material 1) [39]. Interviews consisted of a series of initial questions with follow up questions and probes to explore how multiple level factors (individual, intrapersonal, and organisational influences) could have contributed to their experiences. The study was approved by an Australian university research ethics committee (ethics #H0010875) and follows the COREQ guidelines (Supplementary material 2) for the reporting of qualitative research [40]. All transcripts are available from the first author.

### 2.5. Analysis

An inductive (i.e., data driven), realist thematic analysis was chosen to analyse the lived experience transcripts [41]. A realist thematic analysis assumes an underlying association between internal attitude and representation of it, which is not confined to the context of speech [30]. The process of analysing the transcripts involved several phases and processes [41]. Analysis was conducted by three full time Australian university male researchers (DC, CM, & SP) who developed and implemented the intervention and conducted the training for PC. One full-time male researcher from an American university (HG) and a postgraduate male student (AY) who were independent of this study, but part of the larger project was included in the triangulation process as they attended monthly meetings and were privy to the discussions of the findings and contributed to the feedback. All employed researchers had PhDs in kinesiology research and were experienced in quantitative and qualitative research methods. Before the commencement of coding, all participants were offered the opportunity to read their complete transcripts and make comment for accuracy, correction, or any other issue. No participant availed themselves of this offer.

The initial analysts (DC & SP) involved a read through the transcripts twice to maximise familiarity with the data. Initial codes were then generated systematically for text that appeared relevant in the context of the research question. After all transcripts had been analysed, codes were collated into potential themes by the first and second coder independently. Potential themes were then discussed with the inclusion of a third researcher (CM) to reduce the possibility of personal bias and minimise any interpretative bias. Subsequently, two researchers (HG & AY) were involved in regular discussion of the findings and providing feedback. Potential themes were discussed and reworked with the involvement of all five researchers until key themes and subthemes were generated for the entire data set [30]. Themes which were determined to be irrelevant or ambiguous were discarded from the overall thematic map (Supplementary material 3). For example, one early theme of body image and movement activities was discarded because only one participant commented on this as an issue and no other participant referred to body image issues. Given that data collection was constrained by the study timeline and a limited sample, although this in and of itself is not problematic [32], issues of quantity of data and saturation were addressed by using an analytical process that focused on the notion of conceptual depth [41]. In reporting the findings, the selection of direct quotes from participants emphasised the data richness, depth, diversity, and complexity as evidence of the validity of the data [42]. In cases where ambiguity was thought possible, punctuation was added, and words placed in parentheses to clarify meaning. All results, including those not reported here were presented back to the organisation in a presentation open to all participants as part of the final reporting process.

### 2.6. Rigor

To ensure the quality of this smaller study, we adhered to a checklist criteria for good thematic analysis [42]. These criteria relate to the process of transcription, coding, analysis, and the creation of a written report. Data checking included assessment of the accuracy of transcribed data, checks to ensure all data were treated equally, confirmation that all coded items had been collated, all themes had been checked against the original data set, and stating all assumptions and approaches to thematic analysis [42]. As this study was part of a larger project, the analysts were able to share and discuss their findings with a multidisciplinary group of researchers for feedback. Engaging in this process allowed the analysts to think more deeply about the data. As we believe that there is no single, objective truth, we make no claim to greater accuracy of our findings through the use of researcher triangulation [30]. Nonetheless, we maintain that it allowed us to provide a nuanced account of desk-based workers experiences of having their behaviour choices reduced or nudged to preferred options.

## 3. Results

The initial analysis of all transcripts identified seven lower order themes from which emerged two higher order themes labelled as “facilitation behaviours and feelings” and “dysfunctional behaviours and feelings”. The nomenclature for the themes was derived from researchers' understanding of the literature specific to behaviour change and associated emotions, in particular the conceptualisation that emotions can be facilitative or maladaptive [43],[44] in terms of their mediating effect on individual motivation. The themes are not mutually exclusive but cut across and between different levels of influence: advocacy; acceptance; facilitative burden; loss of control, presenteeism; and reactance. The first theme details how using managers as PC can be facilitative to modify the normative and work cultural influences to implement a behaviour change strategy using passive choice architecture. The second theme describes how individual concerns about new behaviour can generate feelings of psychological discomfort, which impede adherence and compliance to the WHI.

*Theme 1. Facilitating behaviours and feelings*

Sub-theme 1.1. Advocacy

Consistent across participants responses was their positive regard for the WHI and a willingness to be advocates for WHI. This advocacy proved to be a facilitative factor for recruitment of the intervention across the various workforces. This is exemplified by one manager “I saw the advertisement on the intranet of the programme and the results. I thought it was fantastic and wondered why we did not have it.” (Colleen, Workplace A). Another was more direct in securing the intervention for her sector “I attended the health promotion seminar where I heard you speak about some of the preliminary results and was asked by my line manager why it had not been rolled out in my section? We had it the next day.” (Fiona, Workplace A). Moreover, managers' sense of advocacy was also facilitative to employee recruitment “You have a responsibility for looking after your staff in all aspects, and this area was lacking.” (Karen, Workplace C).

Part of the advocacy of the managers was a belief that being a PC fell into their role remit to be responsible for their subordinate's health, spoke of a felt sense of duty for her own health but take on the extra responsibility “So, I felt that this was something that I needed to do, like stand regularly, but also be the role model.” (Heidi, Workplace B). One manager spoke of a how taking on the PC role was a burden because of workload but was also facilitative in terms of creating a positive affective state.

“I can remember telling everybody about it through the day and how it would help them with some of their health issues. I felt good, that I was helping make the office a healthier place for people. But it took some effort, trying to convince people to be involved because it was different. It was extra work, but good work.” (Doris, Workplace E).

This sentiment was often repeated “I don't think people understand how busy I am and the role that I play in this organisation. I enjoy it though being a PC” (Jane, Workplace E). Several other managers agreed, describing positive affective states from being a PC with words such as “I felt good” “expectation” and “no brainer”.

Sub-theme 1.2. Acceptance

Initial responses from the participants to the nudge strategies was positive and contributed to individual motivation to participate in the intervention. Some spoke of the facilitative aspects of the attractiveness of the low intensity movement break activities that could be incorporated into their work routines “I like to think of myself as healthy and the exercises were not that hard. It was a nice feeling to be doing them as part of work” (Colleen, Workplace A). Fiona (Workplace A) was positive towards the intervention content because of the emphasis on movement rather than physical fitness “I liked the movements, that was fun because there was no pressure to be a fitness fanatic, anybody could do them.” Jane (Workplace E) responded similarly to the experience of performing non-exercise physical activity during work “I liked the idea of just walking the stairs as part of work.”

Initially several factors associated with the passive choice architecture enabled compliance. These included: ease of use; “It was simple and easy to do.” (Karen, Workplace C), a reduced mental load “I did not mind it, the prompt to get up and move, I did not have another thing to think about.” (Heidi, Workplace B), and a nudge to help perform the default behaviour “I thought it was easy and not much different to what I thought I normally did, that is stand regularly. I wasn't though, I did not realise I spent so long sitting” (Colleen, Workplace B) or engage in action behaviour “I liked being reminded to take a break...the activities were easy... I was doing lots of them anyway, but not as regular.” (Jane, Workplace E).

Sub-theme 1.3. Facilitative burden

An enabler to implementation was the socio-cultural factor associated with managers in workplaces and the expectations associated with the role. Specifically, participants expressed a consistent organisational level perception of their managerial normative behaviour. Participants across workplaces perceived that taking on the PC role was an extra work burden but accepted the role because it was perceived to be congruent with their status “...so, I take the burden on...but I like that feeling, sort of like I am indispensable to the organisation” (Fiona, Workplace A), the need to ensure completion of tasks “...as a manager, you feel the need to show that you are willing to do the extra (...being a role model), to get things done” (Colleen, Workplace A), or an inability to not complete set tasks “I am not sure I could walk out if something was not done or completed. That's why I volunteered to be a PC” (Jane, Workplace E). This cultural acceptance of a managers' expectations to exceed set work tasks was reflected in their attitudes towards the organisation. This ranged from defensive “I feel protective of the organisation, I hate people criticising the health department” (Fiona, Workplace A), part of identity “I think I will be a little lost when I leave...but in one sense, this job is part of me and I'm part of the job. I know that sounds strange” (Jane, Workplace E), to ownership “I think of this organisation as mine, especially this section that I am in charge of.” (Colleen, Workplace A).

*Theme 2. Maladaptive behaviours and feelings*

Sub-theme 2.1. Negative emotions

Participants expressed negative affective emotions because of the stress associated with convergence of their respective work roles, the complexity of the PC role, and adhering to the default behaviour requirements, “But I was a model for the damn thing...I don't like those feelings, having work to do but having to take a break. It made me tense and somewhat anxious” (Heidi, Workplace B). The feelings of discomfort seem to stem from a feeling that as a role model, managers perceived that they were regularly away from their designated work site and unavailable to their staff.

“I felt uncomfortable being away from my desk, like apprehensive. The others in my group did not seem to mind or at least didn't say anything. Its extra pressure being a supervisor and having to be a PC. If I had my time again, I think I would do things differently, perhaps not being a PC would have changed my feelings. I constantly felt uncomfortable...like uneasy about not being able to do my job and be the PC. Always thinking how I am going to manage every day, the demands on my time were so unexpected...what if someone needs my help and I'm not there?” (Jane, Workplace E)

The connection between heightened negative affective states and the PC roles was common amongst all managers “...but I felt sick, like how am I going to balance everything.” (Colleen, Workplace A). For this manager, there was worry about being able to maintain existing levels of behaviour because of being absent because of a movement break “There were times when I become anxious about having a break and feeling like, what if one of my team needs me, I'm not there.” (Colleen, Workplace A).

Sub-theme 2.2. Control

A sub theme evident in the PC narratives was how the nature of the passive design altered the organisational cultural balance in workplaces by creating an illusion of incongruency between work and health behaviour. Managers not only had to deal with their own individual behaviour change but also the changing group behaviour. Having staff regularly engage in a health behaviour that took them away from their workstations led to a perception of a loss of control over the workplace “It's a big change, you know, letting people stop work and move about. That has never happened before, you know, not having people at their desks.” (Heidi, Workplace B). Some managers spoke to an uncomfortable adjustment associated with their staff engaging in the new health behaviour.

“I would sometimes look up and need to talk to a staff member and they were not there. I'd ask and then be told that they were away having a movement break. At first, I was annoyed, it took a little getting used to.” (Colleen, Workplace A).

For women managerial PCs who withdrew, a perception of a loss of control associated with their held expectations for their own behaviour led to some using external attributions for withdrawing. They used attributions associated with the need to prioritise work productivity over their own individual health behaviour “I felt out of control at times, working and then having a break. Even though I probably needed a break, I felt I needed to get things done.” (Jane, Workplace E), a sense of individual importance “What do they do when they have to ask a question or if the phone rings? Who will answer it? So, I feel tied to the desk.” (Karen, Workplace C), and perfectionism “If I cannot do something at 100 per cent, then I don't do it. I didn't like the change that the prompt caused to me and my work.” (Fiona, Workplace A). Associated with feelings of a loss of control, were perceptions that their behaviour was being surveilled by members of their team “When I first started, I felt everyone was watching me. I didn't like that, like what does she think she is doing?” (Doris, Workplace D). For another, despite the de-emphasised physical activities, they still felt an expectation of perfectionism “but I felt that I had to always be the perfect one, stand up and moving around every hour but get all of my work done caused stress...having other workers seeing me do that” (Heidi, Workplace B). For another, there was a perceived awareness of others watching to ensure that they maintained work standards “I constantly felt uncomfortable...like uneasy about my staff watching me ... checking on me not being able to do my job and be the PC” (Jane, Workplace E).

Sub-theme 2.3. Reactance

The women participants spoke of feelings associated with unpleasant arousal which derived from the constant demand to respond and comply to the default behaviour of regularly standing and moving. This reactance stemmed from constant disruption to workflow “I could not get used to it. ...it seems to disrupt everything. I know I need to stand but being told every hour. I just did not need to be told it.” (Colleen, Workplace A), and interruptions “In the end, I was sick of the interruptions caused by the programme. There needs to be balance. I'm not a person that likes to be told what to do. I choose.” (Colleen, Workplace A). Another manager told of the prompting becoming tiresome and then using workload as an excuse to withdraw “It became a little tiresome to do this because I had to always think about my behaviour, so in the end I just stopped and used the amount of work I had to do as an excuse.” (Doris, Workplace D). Perhaps one manager, Karen (Workplace C), sums up the collective reaction to having respond to the constant nudging after nearly 4 weeks “Look, I have to either get my work done or do physical activity. After a while I chose work because of that f**king prompt ... sometimes I just wanted to smash it when my screen disappeared.”

Sub-theme 2.4. Presenteeism

Emerging from the data were participants' reports of experiencing psychological discomfort associated with a self-awareness of how the default behaviour was incongruent with the normative behavioural contract for their role. This discomfort morphed into feelings of presenteeism.

“I hated it, hated it. It made me feel so bad, like I was stealing because I was not working at my desk, or someone might notice that I wasn't at my desk and ask where I was. I don't like those feelings, having work to do but having to take a break. It made me tense and somewhat anxious. I think it's a great idea and we do sit for long periods, but not for people like me. It does not work. No. I could not participate any longer.” (Fiona, Workplace A).

Issues of perceived surveillance contributed to feelings of presenteeism in some managers, Doris (Workplace D) remarked “I felt everyone was watching me. I didn't like that, like what does she think she is doing?” Heidi (Workplace B) mentioned that fears of surveillance led to enacting change in her behaviour, whereby only certain less obvious or ambiguous movement activities were selected to try and maintain a perception of adherence to normative behaviour expectations while trying to adhere to the default normative behaviour “I did try and do some of the less obvious activities like standing while talking on the phone”. Another reported using external attributions connected to their role expectations and importance as too greater cost for the health benefit.

“My job is too important for me to be off doing exercise, it's not what I am paid for and not what others expect of me... I know sitting for long periods is not good for me, my back aches, but I want to get home at a reasonable hour, so I cannot stop. I'm not paid to stop. I feel some pressure not to stop.” (Karen, Workplace C).

## 4. Discussion

The aim of our research was to explore the lived experiences of women managers who acted as PCs but unexpectedly withdrew from a nudge-based WHI designed to influence their decisions about work behaviour choice. Understanding the nature of their perceived exchanges was relevant to disentangle the key individual and situational moderators associated with using nudges to influence behaviour choices. The first finding is an artefact of the purposive sample, only a quarter of women managers who acted as PCs had experienced an exchange which caused them to withdraw from the study. The bias in the sample is reflective of some literature, that when compared to women's decisions, the decisions of men can be influenced by their attitude whereas in contrast, women were more strongly influenced by subjective norm and perceived behavioural control [29]. Thus, for some PCs, the loss of choice through the use of a passive nudge strategy and being nudged to choose workplace behaviour that was not reflective of existing work role expectations resulted in a negative emotional exchange. Subsequently, they withdrew from the intervention. This explanation is supported by our findings which revealed two themes and their subsequent sub-themes which reflect assessments of the personal exchanges perceived as a result of being exposed to nudge strategies [27].

The facilitative theme and its sub-themes findings provide evidence to support previous literature and research that individuals experience a positive exchange when exposed to nudge strategies designed to influence the workplace behaviour choices [17],[22]. The parsimonious nature of nudging provided acceptance of the nudge strategies. Moreover, there was a facilitative effect whereby managers advocated and actively recruited participants for the intervention. Although there was an element that being a PC was perceived as part of the work role [36]. Nudges are a popular strategy to help people make changes to their behaviours and our findings support the contention that they can be employed in workplaces [17]. Moreover, our findings align with the literature that nudges reduce individuals' perceptions of cognitive load associated with choosing alternative behaviour choice [17],[45],[46], thus adding to the perceived positive exchange of being exposed to them. This is pertinent as workplaces are by their nature, busy with workers following habitual work routines. Changing these routines presents a possible negative exchange where individual workers have to continue to remind themselves to engage in a new behaviour. Our results suggest that workers perceived a positive exchange from being exposed to nudges is congruent with the literature which describes the benefits of using a passive choice architecture and normative-based strategies [17],[47],[48].

Despite the evidence of a positive exchange, this only lasted a short period for the PCs who withdrew. Our findings indicate that these managers perceived a negative exchange in terms of perceived behavioural monitoring by co-workers which evoked feelings of facilitative burden. These feelings seemed to emanate from the decision to choose alternative work behaviours which increase perceptions of co-workers and self-monitoring. This monitoring resulted in judgements of congruency and expectations associated with work role behaviour. Consistent was the mediating variable of ownership, with feelings of strong ownership moderating to the decision to maintain alternative behaviour choice [27].

## 5. Conclusions

To the best of our knowledge, this is the first evidence that individuals can experience positive and negative exchanges from being exposed to certain nudge strategies. Our findings illustrate how decision-making associated with being nudged to choose alternative healthier work behaviours are tangled with the workplace environment, workers' emotional connections to their work identities, and the causal mechanisms associated with compliancy behaviours. Together, our findings contain several salient issues for the implementation of passive choice and social norms as nudges to increase movement break behaviour in workplaces. First, the use of passive choice architecture as a framework for introducing new workplace health behaviour has ecological validity. Second, when behavioural choice outcomes are public, such as nudging workers to participate in hourly movement breaks, self-awareness, and perceived monitoring by others of the behaviour choice seems to be moderating variables for the use of nudge strategies. The issue of changing behaviour within the emotional-laden and social context of work needs further investigation to determine the efficacy of different nudge strategies to untangle socio-cultural aspects of the work environment [49].

Study limitations must be acknowledged. Our purposive sample was small and restricted to those who withdrew early from the intervention. We only collected their perceptions and have no alternative data from those who adhered to their alternative behavioural decisions to rebut our findings. Our findings are restricted only to post hoc withdrawal memories, as participants were not required to keep diaries of significant experiences. Nonetheless, memories do form the input for behaviour [50] and the findings do support the notion that when behaviour exchanges result in negative affect, withdrawal from the change process is inevitable. Our findings provide data for a largely unrepresentative group, that is, PCs who withdrew from a nudge-based WHI, and how their experiences of positive and negative exchanges resulted in their decision to withdraw their involvement from the WHI.
