# Supplementary material for: Peer champions responses to nudge-based strategies designed to reduce prolonged sitting behaviour: Lessons learnt and implications from lived experiences of non-compliant participants
Source: AIMS Public Health. 2022 Jul 12;9(3):574–88. doi: 10.3934/publichealth.2022040 (PMC9581742; doi:10.3934/publichealth.2022040)
Supplement: Supplementary file 1 [file publichealth-09-03-040-s001.pdf]

AIMS Public Health

AIMS Public Health, 9(3): 574–588.
DOI: 10.3934/publichealth.2022040
Received: 24 March 2022
Revised: 28 June 2022
Accepted: 05 July 2022
Published: 12 July 2022

http://www.aimspress.com/journal/aimsph

*Research article*

# Peer champions responses to nudge-based strategies designed to reduce prolonged sitting behaviour: Lessons learnt and implications from lived experiences of non-compliant participants

**P. Dean Cooley[1], Casey P. Mainsbridge[1,*], Vaughan Cruickshank[1], Hongwei Guan[2], Anjia Ye[1] and Scott J. Pedersen[1]**

[1] College of Arts, Law and Education, University of Tasmania, Launceston, Tas, Australia
[2] Ithaca College, Ithaca, New York, USA

* **Correspondence:** Email: Casey.Mainsbridge@utas.edu.au.

## Supplementary

**Supplementary material 1: Guide for face-to-face & telephone interviews.**

1. How did you and your workgroup become to be involved in this intervention?
2. Thinking back to the first experiences with the computer application, what were your initial responses to:
   a. the activities
   b. the prompts
   c. the movement breaks
   Prompt
   Any other aspects you would like to comment on?
3. If we can, I would like to discuss your impressions of the intervention.
   a. Can you describe your motivation at the start to engage in movement breaks?
   b. Could you describe aspects of the intervention that you liked?
   Now if you like can we discuss your work role in the organisation and the decision to be a peer champion?

4. Thinking about this organisation, what are your feelings about:
   a. The organisation
   b. Your work role
   c. Can you describe if there was a connection between your work role and being a peer champion
5. Can you describe your thinking about why you volunteered to be a peer champion?
   a. Can you describe any emotions associated with being a manager and being a peer champion?
   b. Did those emotions influence your behaviour? How so, can you describe the connection?
   I'd like you to think about your work routines and behaviours and the requirement to participate in the default health behaviour.
6. Can you give an example of symmetry in your work roles/ peer champion and the default health behaviour?
   a. Can you describe any emotions?
   b. Can you describe any benefits?
7. Can you give an example of conflict in your work roles/ peer champion and the default health behaviour?
   a. Can you describe any emotions?
   b. Can you describe any barriers?
   If we can I would like to gain an understanding of your initial reaction to the prompt and loss of your computer screen to help you engage in the regular movement breaks.
8. I would like you to think about your initial reaction (emotions, thoughts) to parts of the programme at the start … say the first week?
   a. The prompt?
   b. Losing your screen?
   c. The activities?
   d. Participating in movement breaks during work?
9. I would like you to think about your reaction (emotions, thoughts) to parts of the programme in the lead up to you deciding to withdraw from the programme at the start:
   a. The prompt
   b. Losing your screen
   c. The activities
   d. Participating in movement breaks during work
10. Were there any aspects about your work role, the work situation or other things in the workplace surroundings that influenced your decision to withdraw?
    a. How did those factors you just described impact on you?
    b. Did you feel if other people in your work area around you were aware of your movement breaks?
    c. Did being a manager make any difference?
11. Can you describe any changes in your work performance while in the programme?
    a. Any effect on productivity?
    b. Any effect on ability to concentrate?
    c. Availability?

**Supplementary material 2: COREQ checklist.**

| **Domain 1. Research team and reflexivity** | | **Location in manuscript** |
|---|---|---|
| **Personal characteristics** | | |
| 1. Interviewer/facilitator Which author/s conducted the interview or focus group? | VC | Method<br>Page 7<br>Line 4 |
| 2. Credentials<br>What were the researcher's credentials? | PhD, B.Ed | Method<br>Page 7<br>Line 4 |
| 3. Occupation of interviewer | Academic | Title page |
| 4. Gender | Male | Method<br>Page 7<br>Line 4 |
| 5. Experience & training | The interviewer had completed numerous interviews for other qualitative studies | Method<br>Page 7<br>Line 4–5 |
| **Relationship with participants** | | |
| 6. Relationship established: Was a relationship established Was a relationship established prior to study commencement? | Interview researcher was independent of the initial research | Method:<br>Page 7<br>Line 4–5 |
| 7. Participant knowledge of the interviewer What did the participants know about the researcher? e.g., personal goals, reasons for doing the research | Participants understood the nature and purpose of the study, understood ethical approval carried over as part of the larger study. Participants understood the interviewer was independent of the larger study | Method:<br>Page 7<br>Line 4–5 |
| 8. Interviewer characteristics What characteristics were reported about the interviewer/facilitator? e.g., Bias, assumptions, reasons, and interests in the research topic | VC holds a tenured role as a researcher/academic.<br>No other information given to participants. Researcher not part of the initial study | Method:<br>Page 7<br>Lines 4–5 |

| **Domain 2. Study design** | | **Location in manuscript** |
|---|---|---|
| **Theoretical framework** | | |
| 9. Methodological orientation and Theory What methodological orientation was stated to underpin the study? e.g., grounded theory, discourse analysis, ethnography, phenomenology, content analysis | reflexive thematic analysis with deductive coding | Method Page 5 Lines 1–6 |
| 10. Sampling How were participants selected? e.g., purposive, convenience, consecutive, snowball | Purposive sampling. Recruit via email after participant had withdrawn from a larger study | Method Page 6 Lines 6–11 |
| 11. Method of approach How were participants approached? e.g., face-to-face, telephone, mail, email | Email invitation to participate and share experiences | Method Page 6 Line 1–2 |
| 12. Sample size How many participants were in the study? | Six | Method Page 5 Line 19 |
| 13. Non-participation How many people refused to participate or dropped out? Reasons? | All agreed to participate in the study | Method Page 6 Line 1 |
| **Setting** | | |
| 14. Setting of data collection Where was the data collected? e.g., home, clinic, workplace | Data was collected in face-to-face interviews in participants' office spaces and through zoom meetings | Method Page 7 Line 9–17 |
| 15. Presence of non-participants Was anyone else present besides the participants and researchers? | No other participants present | Method Page 7 Line 9–17 |
| 16. Description of sample What are the important characteristics of the sample? e.g., demographic data, date | Age range 30-45 6 women who were full time employees. All participants were supervisors of small teams of employees | Table 1 |
| **Data Collection** | | |
| 17. Interview guide Were questions, prompts, guides provided by the authors? Was it pilot tested? | Interviews were semi-structured using a schedule of questions and suggested prompts (Table 2); follow up questions were allowed. | Supplementary Table 2 |
| 18. Repeat interviews Were repeat interviews carried out? If yes, how many? | One repeat interview with each participant was completed with all participants | Method: Page Line 12 |

*Continued on next page*

| **Domain 2. Study design** | | **Location in manuscript** |
|---|---|---|
| **Theoretical framework** | | |
| 19. Audio/visual recording<br>Did the research use audio or visual recording to collect the data? | The face to face and video semi-structured interviews were digitally audio recorded using a laptop. This allowed audio recording of face-to-face and video interviews by the same method | Method<br>Page 7<br>Line 19–20 |
| 20. Field notes<br>Were field notes made during and/or after the interview or focus group? | Additional field notes were made | Method<br>Page 7<br>Line 15 |
| 21. Duration What was the duration of the interviews or focus group? | Semi structure interview times ranged from 58 minutes to 84 minutes | Method<br>Table 1 |
| 22. Data saturation<br>Was data saturation discussed? | No discussion before analysis but agreement reached for ant of narrative lines to be used as evidence | Method<br>Page 9<br>Line 8 |
| 23. Transcripts returned Were transcripts returned to participants for comment and/or correction? | Participants were given the opportunity to have personal transcripts returned but none availed themselves | Method<br>Page 9<br>Line 11–13 |

| Domain 3. Analysis and findings | | Location in manuscript |
|---|---|---|
| **Data analysis** | | |
| 24. Number of data coders How many data coders coded the data? | 5 data coders, two initial coders with three reviewers | Method<br>Page 8<br>Lines 20–25 |
| 25. Description of the coding tree<br>Did authors provide a description of the coding tree? | Yes<br>Deductive open coding Process described in the methods section | Method<br>Page 9<br>Lines 16 |
| 26. Derivation of themes<br>Were themes identified in advance or derived from the data? | Themes were derived from the data. | Method<br>Page 9<br>Lines 18 |
| 27. Software What software, if applicable, was used to manage the data? | Microsoft word, excel, NIVIVO | Method<br>Page 7<br>Lines 19–20 |
| 28. Participant checking<br>Did participants provide feedback on the findings? | All results were presented as in a collective in a presentation open to all participants. | Method<br>Page 9<br>Lines 9–13 |
| **Reporting** | | |
| 29. Quotations presented Were participant quotations presented to illustrate the themes / findings? Was each<br>quotation identified? e.g., participant number | Yes, specific examples of comments were supplied with direct quotes attributed to anonymised participant. | Findings section<br>Page 10 onwards |
| 30. Data and findings consistent<br>Was there consistency between the data presented and the findings? | Yes | Method<br>Page 10<br>Lines 2–12 |
| 31. Clarity of major themes<br>Were major themes clearly presented in the findings? | Yes – two major themes emerged from the analysis | Findings<br>Pages 10 onwards |
| 32. Clarity of minor themes Is there a description of diverse cases or discussion of minor themes? | Minor themes are discussed in the manuscript | Findings<br>Pages 10 onwards |

**Supplementary material 3: Coding tree.**

**Theme 1: Facilitating behaviours and feelings.**

1. Advocacy
    a. Active recruitment
    b. Expected role
2. Acceptance
    a. Ease of use
    b. Fun
    c. Ergonomically sound

3. Facilitative burden
    a. Normative behaviour
    b. Ownership and identity

**Theme 2: Maladaptive behaviours and feelings.**

1. Negative affect
    a. Increased awareness of convergence of work and PC role
    b. Awareness of role behaviour violation
    c. Absent from designated area
    d. Physical demand vs employee need
2. Control
    a. Psychological discomfort arising from:
        i. Incongruency with work role expectations
        ii. Heightened awareness of surveillance of self and by others
3. Reactance
    a. Unpleasant arousal emanating from:
        i. Interruption to workflow
        ii. Preoccupation with behaviour
4. Presenteeism
    a. Heighten feelings of being watched
    b. External attribution - job importance
    c. Altered behaviour choice

AIMS Press

© 2022 the Author(s), licensee AIMS Press. This is an open-access article distributed under the terms of the Creative Commons Attribution License (http://creativecommons.org/licenses/by/4)
